# Cell-Type-Specific Effects of Silibinin on Vitamin D-Induced Differentiation of Acute Myeloid Leukemia Cells Are Associated with Differential Modulation of RXR**α** Levels

**DOI:** 10.1155/2012/401784

**Published:** 2012-05-20

**Authors:** Rina Wassermann, Victoria Novik, Michael Danilenko

**Affiliations:** Department of Clinical Biochemistry, Faculty of Health Sciences, Ben-Gurion University of the Negev, P.O. Box 653, 84105 Beer-Sheva, Israel

## Abstract

Plant polyphenols have been shown to enhance the differentiation of acute myeloid leukemia (AML) cells induced by the hormonal form of vitamin D_3_ (1**α**,25-dihydroxyvitamin D_3_; 1,25D). However, how these agents modulate 1,25D effects in different subtypes of AML cells remains poorly understood. Here, we show that both carnosic acid (CA) and silibinin (SIL) synergistically enhancd 1,25D-induced differentiation of myeloblastic HL60 cells. However, in promonocytic U937 cells, only CA caused potentiation while SIL attenuated 1,25D effect. The enhanced effect of 1,25D+CA was accompanied by increases in both the vitamin D receptor (VDR) and retinoid X receptor alpha (RXR**α**) protein levels and vitamin D response element (VDRE) transactivation in both cell lines. Similar increases were observed in HL60 cells treated with 1,25D + SIL. In U937 cells, however, SIL inhibited 1,25D-induced VDRE transactivation concomitant with downregulation of RXR**α** at both transcriptional and posttranscriptional levels. These inhibitory effects correlated with the inability of SIL, with or without 1,25D, to activate the Nrf2/antioxidant response element signaling pathway in U937 cells. These results suggest that opposite effects of SIL on 1,25D-induced differentiation of HL60 and U937 cells may be determined by cell-type-specific signaling and transcriptional responses to this polyphenol resulting in differential modulation of RXR**α** expression.

## 1. Introduction

Acute myeloid leukemia (AML) is a hematologic cancer which results from blocked differentiation of hematopoietic stem and/or progenitor cells due to various genetic and epigenetic errors and is characterized by the uncontrolled proliferation of myeloid blasts. The standard approach for AML treatment is chemotherapy with cytarabine and anthracyclines; however, even after aggressive chemotherapy about 75% of AML patients relapse within 2 years of remission [1, 2]. Recently, several targeted cytotoxic approaches to treat AML have been developed, for example, the use of kinase and histone deacetylase inhibitors [3], but such strategy is difficult to apply to AML, since the molecular lesions in AML are highly heterogeneous.

Differentiation therapy is an alternative or complementary treatment of AML which aims at inducing maturation of poorly differentiated leukemic blasts. The hormonal form of vitamin D_3_ (1*α*,25-dihydroxyvitamin D_3_) is a strong differentiation inducer which has potential for the treatment of AML [4]. However, at concentrations capable of differentiating AML cells in culture 1,25D may cause fatal hypercalcemia *in vivo*. Many low-calcemic vitamin D analogs have been synthesized to date [5], but none has yet been shown to be appropriate for human use at concentrations required to induce differentiation of AML blasts. One way to overcome this problem is to combine low concentrations of 1,25D with other compounds which would enhance its anticancer, but not toxic, effects [6]. We have previously shown that carnosic acid (CA), the major phenolic diterpene of rosemary, potentiates the differentiation effects of 1,25D in AML cell lines representing different developmental blocks in myeloid differentiation, such as myeloblastic (HL60) [7–9], promyelocytic (NB4) [10], and (myelo)monocytic (U937, OCL-AML3, MOLM-13, THP-1) [9–12] leukemia cells, as well as in leukemic blasts derived from patients with AML [11]. Furthermore, combined treatments with either CA or CA-rich rosemary extract and different vitamin D derivatives resulted in enhanced differentiation and growth arrest of WEHI-3B D^−^murine myelomonocytic leukemia cells *in vitro* and—in case of rosemary extract—in cooperative antileukemic effects in syngeneic mouse AML models *in vivo*, without inducing hypercalcemia [13, 14]. Another plant polyphenol, the flavonolignan silibinin (SIL) found in milk thistle, was also demonstrated to potentiate the differentiation effect of 1,25D in HL60 and OCL-AML3 cells [7, 12, 15]; however, this polyphenol tended to attenuate differentiation in U937 and THP-1 cells [16]. In addition, SIL exerted both potentiating and inhibitory effects on the differentiation of leukemic blast samples obtained from different patients with AML [17].

In this study we attempted to clarify the nature of the opposite effects of SIL on 1,25D-induced differentiation of HL60 and U937 cells using CA for comparison. We found that the differentiation-enhancing effect of the 1,25D/SIL combination in HL60 cells was associated with upregulation of vitamin D receptor (VDR) and retinoid X receptor (RXR*α*) levels compatible with increased transactivation of the vitamin D response element (VDRE). On the other hand, the inhibitory effect of SIL on 1,25D-induced differentiation of U937 cells was accompanied by downregulation of RXR*α* expression and attenuated VDRE transactivation.

## 2. Materials and Methods

### 2.1. Chemicals, Antibodies, and Plasmids

 Tissue culture media and reagents were from Invitrogen (Grand Island, NY), Biological Industries (Beit Ha'Emek, Israel), and HyClone (Logan, UT). Carnosic acid was purchased from Alexis Biochemicals (Läufenfingen, Switzerland). 1,25D was a gift from Dr. Andrzej Kutner (Pharmaceutical Research Institute, Warsaw, Poland). Silibinin, cycloheximide, tert-butylhydroquinone (tBHQ), and DMSO were from Sigma (Rehovot, Israel). Stock solutions of CA (10 mM), SIL (30 mM), 1,25D (0.25 mM), and tBHQ (30 mM) were prepared in absolute ethanol. The precise concentrations of 1,25D in stock solutions were verified spectrophotometrically at 264 nm (*ε* = 19,000). The antibodies against NQO1 (C-19), VDR (C-20), RXR*α* (D-20), and TrxR1 (H-270) were from Santa Cruz Biotechnology Inc. (Santa Cruz, CA). Calreticulin antibody (PA3-900) was from Affinity BioReagents (Goden, CO). Peroxidase-conjugated donkey anti-rabbit and donkey anti-goat IgG were from Jackson ImmunoResearch Laboratories Inc. (West Grove, PA). The 4xARE-Luc reporter construct containing four tandem repeats of the antioxidant response element (ARE) sequence from the glutathione S-transferase Ya subunit was a gift from Dr. M. Hannink (University of Missouri, Columbia, MO) [18]. The VDREx6-Luc reporter construct containing a 6-fold direct repeat 3 (DR3) sequence was kindly provided by Dr. L.P. Freedman (Memorial Sloan-Kettering Cancer Center, New York, NY, USA). *Renilla* luciferase expression construct (pRL-null vector) was purchased from Promega (Madison, WI) and served as an internal transfection standard.

### 2.2. Cell Culture, Treatment, and Enumeration

HL60-G cells, subcloned from HL60 human myeloblastic leukemia cells [19], and U937 human myelomonocytic leukemia cells (American Type Culture Collection, Rockville, MD) were grown in RPMI 1640 medium supplemented with 10% heat-inactivated fetal calf serum or bovine serum in a humidified atmosphere of 95% air and 5% CO_2_, at 37°C. Cell cultures were passaged two to three times weekly to maintain a log phase growth. For experiments, cells were seeded in 6-well plates (Greiner Bio-One GmbH, Solingen, Germany) at densities indicated in legends to figures followed by the addition of vehicle (0.1% ethanol), 1,25D (1 nM), polyphenols, or their combinations under dim lighting conditions. Cell cultures were then incubated, as described above, for the indicated time periods. 1,25D alone at the higher concentration of 100 nM was used as the positive control. Cell numbers and viability were estimated on the basis of trypan blue exclusion by counting in Vi-Cell XR cell viability analyzer (Beckman Coulter Inc., Fullerton, CA).

### 2.3. Determination of Markers of Differentiation

Aliquots of 1 × 10^6^ cells were harvested, washed twice with phosphate-buffered saline (PBS), and suspended in 10 *μ*L 1× PBS. The cell suspensions were incubated for 45 minutes at room temperature with 0.3 *μ*L MO1-FITC and 0.3 *μ*L MY4-RD-1 to analyze the expression of the cell surface markers CD11b and CD14, respectively. The cells were then washed three times with ice-cold 1× PBS and resuspended in 1 mL of PBS. Analysis was performed using Cytomics FC500 flow cytometer equipped with CXP software (Beckman Coulter). Isotypic mouse IgG1 was used to set threshold parameters.

### 2.4. Quantitative Reverse Transcription-Polymerase Chain Reaction (qRT-PCR)

 Total RNA was extracted from cells with the PerfectPure RNA Tissue Kit (5PRIME, Gaithersburg, USA) and cDNA was prepared with Verso cDNA kit (ABgene, Epsom, UK), according to the manufacturer's instructions. The following primers were used for the determination of CD11b, CD14, and RXR*α* mRNA expression: CD11b, forward primer (5′-CTGTCTGCCAGAGAATCCAGTG-3′), reverse primer (5′-GAGGTGGTTATGCGAGGTCTTG-3′); CD14, forward primer (5′-GCCCTTACCAGCCTAGACCT-3′), reverse primer (5′-CCCGTCCAGTGTCAGGTTAT-3′); RXR*α*, forward primer (5′-CAAACATGGGGCTGAACC-3′), reverse primer (5′-AAGTGTGGGATCCGCTTG-3′); ARP0, forward primer (5′-AGATGCAGCAGATCCGCAT-3′), reverse primer (5′-GTGGTGATACCTAAAGCCTG-3′). cDNA samples (7 *μ*L) were diluted ninefold, mixed with the specific primers (0.2 mM) and ABsolute Blue SYBR Green ROX Mix (ABgene, Epsom, UK) was then added to the reaction mixture. Reactions were carried out in the Rotor-Gene Real-Time PCR machine (Corbett-Research, Northlake, Australia). Standard cycling conditions for this instrument were 15 min initial enzyme activation at 95°C then 35 cycles as follows: 10 sec at 95°C, 15 sec at the annealing temperature, and 15 sec at 72°C. The results were normalized by ARP0 mRNA content and quantified using the 2^−ΔΔC^_T_ method.

### 2.5. Preparation of Whole Cell Extracts and Western Blot Analysis

Cells were lysed in ice-cold lysis buffer containing 50 mM, HEPES (pH 7.5), 150 mM NaCl, 10% (v/v) glycerol, 1% (v/v) Triton X-100, 1.5 mM EGTA, 2 mM sodium orthovanadate, 20 mM sodium pyrophosphate, 50 mM NaF, 1 mM DTT, and 1 : 50 Complete Protease Inhibitors Cocktail (Roche Molecular Biochemicals, Mannheim, Germany) and centrifuged at 20,000 ×*g*, 10 min, 4°C. Supernatant samples (30 *μ*g protein) were subjected to SDS-PAGE and then electroblotted into nitrocellulose membrane (Whatman, Dassel, Germany). The membranes were blocked with 5% milk for 2 h and incubated with primary antibodies overnight at 4°C followed by incubation with HRP-conjugated secondary antibodies for 2 h. The protein bands were visualized using the Western Lightning Chemiluminescence Reagent Plus (PerkinElmer Life Sciences, Inc., Boston, MA). The blots were stripped and reprobed for the constitutively present protein, calreticulin, which served as the loading control. The optical density (OD) of protein bands was quantitated using Image Gauge 3.11 software (Fuji Photo Film Co., Tokyo, Japan). OD values for each protein were normalized to calreticulin and are displayed beneath each protein band.

### 2.6. Transient Transfection and Reporter Gene Assay

HL60 cells were transiently cotransfected with 4xARE-luc reporter plasmid (1.35 *μ*g) and *Renilla *luciferase vector (0.15 *μ*g) or VDREx6-luc reporter plasmid (0.95 *μ*g) and *Renilla* luciferase vector (0.05 *μ*g) using Microporator (Digital Bio Technology, Seoul, Korea) under the following conditions: 1 pulse, 1400 Volts, pulse width 30 msec. U937 cells were transiently cotransfected with 4xARE-luc or VDREx6-luc reporter plasmids (0.8 *μ*g) and *Renilla* luciferase vector (0.2 *μ*g) using JetPEI Reagent (POLYplus-Transfection, Illkirch Cedex, France), according to the manufacturer's instructions. Transfected cells were exposed to the indicated treatments for 24 h followed by measurement of luciferase activity using the Dual Luciferase Reporter Assay system (Promega, Medison, WI, USA). The data are presented as the normalized ratios of firefly luciferase to *Renilla* luciferase activity (relative luminescence units, RLU).

### 2.7. Statistical Analysis

The significance of the differences between the means of the various subgroups was assessed by unpaired two-tailed Student's *t*-test. Two compounds (*A* and *B*) were considered to show enhancement in the particular experiment if the effect of their combination (*AB*) was larger than the sum of their individual effects (*AB* > *A* + *B*), the data being compared after subtraction of the respective control values from *A*, *B*, and *AB* [8]. The statistical analysis was performed with the GraphPad Prism 5.0 Program (Graph-Pad Software, San Diego, CA). Data are presented as the mean ± SE. *P* < 0.05 was considered statistically significant.

## 3. Results

### 3.1. Silibinin Potentiates 1,25D-Induced Differentiation in HL60 Cells While Attenuating It in U937 Cells

**Figure 1:**
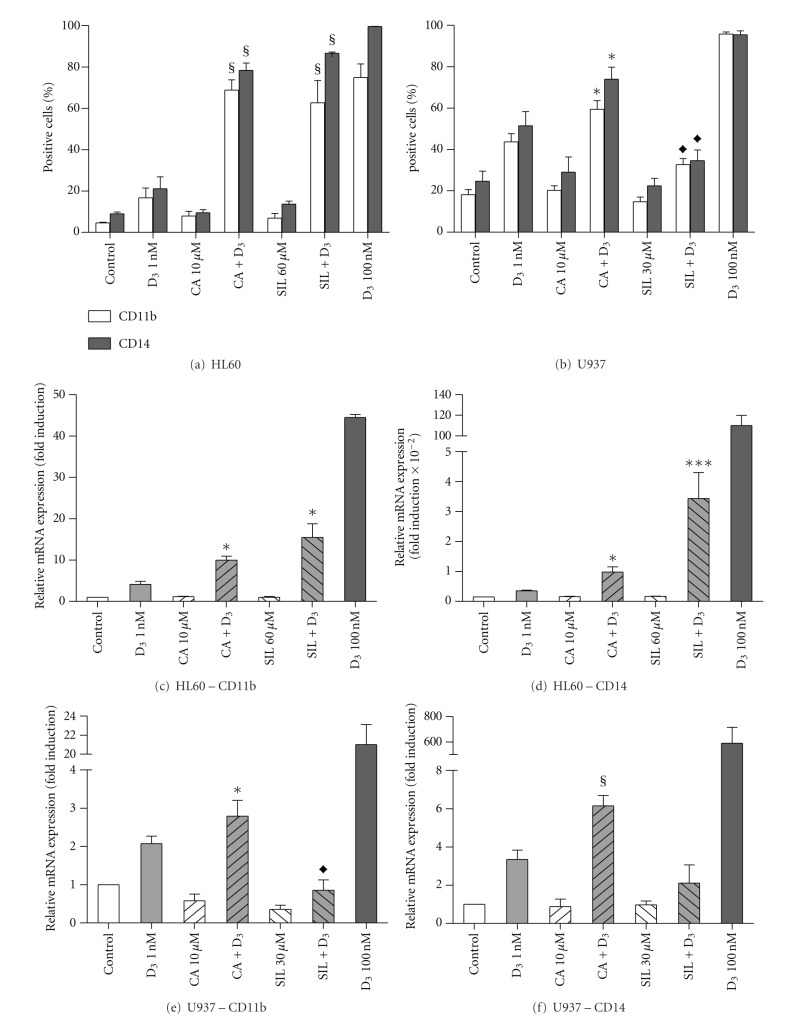
Carnosic acid enhances while silibinin differentially affects 1,25D-induced differentiation of HL60 and U937 cells. Cells were incubated at 4 × 10^4^ cells/mL (a), (b) or 5 × 10^4^ cells/mL (c-f) with 0.1% ethanol (vehicle control) or the indicated test agents, alone or in combination, for 96 h (a), (b) or 24 h (c-f). CD11b and CD14 expression was then determined by flow cytometry (a), (b) or qRT-PCR (c-f). The data are the means ± SE of 5 (a), (b) or 3 (c-f) independent experiments. *P < 0.05; ^§^ *P* < 0.01, or ***P < 0.001, combination *versus* sum of single agents for enhancing effects. ^♦^ *P* < 0.05, combination versus 1 nM 1,25D alone for inhibitory effects.

We first compared the effects of CA and SIL on the expression of two cell surface markers of myeloid differentiation, CD11b and CD14, induced by a low concentration of 1,25D (1 nM) in HL60 and U937 cells (Figure 1). To exclude potential interference of reagent cytotoxicity in the determination of the vitamin D receptor expression and activity, as described in the following sections, we used noncytotoxic concentrations of the two polyphenols: 10 *μ*M CA for both cell lines [7, 8, 20], 60 *μ*M SIL for HL60 cells [7, 16, 17, 20], and 30 *μ*M SIL for U937 cells. Under these conditions, cell viability was maintained at 95–98% throughout the course of the experiments performed here. Concentrations of SIL higher than 30 *μ*M caused a dose-dependent decrease in the viability of U937 cells (up to ~20% dead cells at 60 *μ*M SIL; data not shown). The addition of CA to 1,25D not only synergistically increased the percentage of CD11b- and CD14-positive HL60 and U937 cells, as measured by flow cytometry following 96 h treatment (Figures 1(a), 1(b)), but also markedly enhanced the mRNA expression of these markers after 24 h (Figures 1(c), 1(d), 1(e), and 1(f)), as compared to 1,25D alone. These potentiating effects of CA were more pronounced in HL60 cells than in U937 cells. On the other hand, while being even a stronger differentiation enhancer than CA in HL60 cells (Figures 1(a),1(c), and 1(d)), SIL appreciably reduced 1,25D-stimulated expression of CD11b and CD14 in U937 cell, as determined by both flow cytometry (Figure 1(b)) and qRT-PCR (Figures 1(e), 1(f)).

### 3.2. Silibinin Cooperates with 1,25D to Induce Growth Arrest in HL60 Cells but Not in U937 Cells

**Figure 2:**
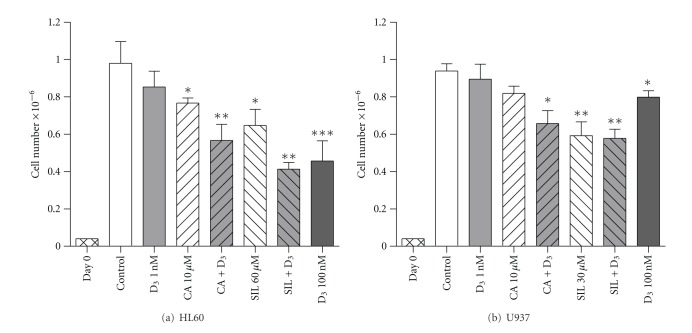
Carnosic acid and silibinin, alone and in combination with 1,25D, inhibit proliferation of HL60 and U937 cells. Cells were seeded at 4 × 10^4^ cells/mL (day 0) and treated with test agents, for 96 h, as described in legend to Figure 1. Following incubation, cells were counted using the trypan blue exclusion assay. The numbers of viable cells are presented as the means ± SE of at least 5 independent experiments. *P < 0.05; **P < 0.01, or ***P < 0.001, treatments *versus* control.

In parallel with flow cytometric determination of CD11b and CD14 expression (Figures 1(a) and 1(b)), cells from the same wells were enumerated to examine the effects of 1,25D and polyphenols on cell proliferation and viability. As shown in Figure 2(a), both CA and SIL administered alone for 96 h significantly decreased the number of proliferating HL60 cells while 1 nM 1,25D alone had a minor effect. However, the addition of 1,25D to either of the two polyphenols resulted in a marked cooperative inhibition of cell growth comparable to that produced by high-dose 1,25D (100 nM). U937 cells exhibited lower sensitivity to the antiproliferative effect of 1,25D, as compared to HL60 cells. CA produced similar effects in both cell lines, whereas SIL caused somewhat stronger growth inhibition in U937 even at a 2-fold lower concentration than used in HL60 cells (Figure 2(b) *versus* Figure 2(a)). Interestingly, while treatment with the 1,25D/CA combination resulted in a more pronounced reduction in U937 cell numbers than that exerted by single compounds, the antiproliferative effect of SIL was not significantly altered by 1,25D (Figure 2(b)). In the previous experiments, no treatment tested significantly affected cell viability (93–96% in all samples). These findings are consistent with our previous data showing antiproliferative effects of CA and SIL, alone and in combination with different vitamin D derivatives, in murine and human AML cell lines [8, 9, 13, 14, 20, 21].

### 3.3. Silibinin Potentiates 1,25D-Induced VDRE Transactivation in HL60 Cells While Inhibiting It in U937 Cells: Association with RXR*α* Protein Levels

**Figure 3:**
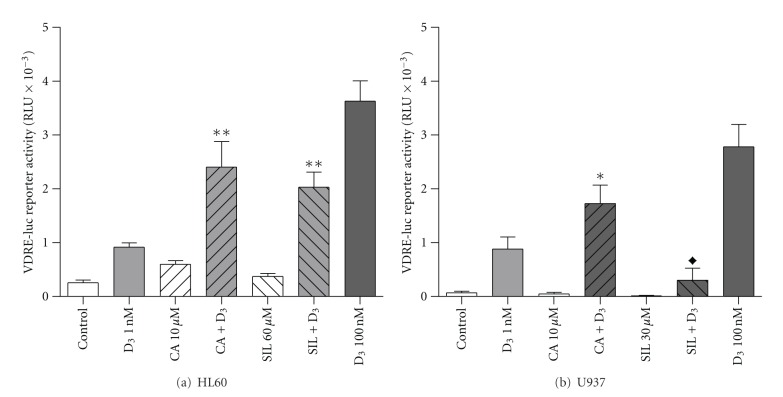
Carnosic acid enhances while silibinin differentially affects 1,25D-induced VDRE transactivation in HL60 and U937 cells. Cells (5 × 10^5^ cells/ml) were transiently transfected with VDRE×6-luc and *Renilla* luciferase reporter constructs followed by treatment with 0.1% ethanol (control) or indicated test agents for 24 h. The relative VDREx6-luc activity (means ± SE) was calculated from the data of 3 individual experiments performed in triplicate. *P < 0.05 and **P < 0.01, combination *versus* sum of single agents for enhancing effects. ^♦^ *P* < 0.05, combination versus 1 nM 1,25D alone for inhibitory effects.

To examine the mechanism underlying the differential effects of SIL on 1,25D-induced differentiation in HL60 and U937 cells we tested the hypothesis that, similar to CA, SIL promotes the functional activation of the vitamin D receptor by 1,25D in HL60 cells while, in contrast to CA, attenuating it in U937 cells. To this end, we first compared the effects of CA and SIL on 1 nM 1,25D-induced VDRE transactivation in transiently transfected HL60 and U937 cells using VDRE-Luc reporter gene assay. As expected, both CA and SIL markedly enhanced 1,25D-stimulated transcription from VDRE in HL60 cells (Figure 3(a)). A similar potentiating effect of CA was observed in U937 cells; however, SIL significantly reduced VDRE transactivation by 1,25D in this cell line (Figure 3(b)). The above differential effects of CA and SIL on VDRE activation correlated with their corresponding modulation of 1,25D-induced differentiation in the two cell lines (see Figure 1).

**Figure 4:**
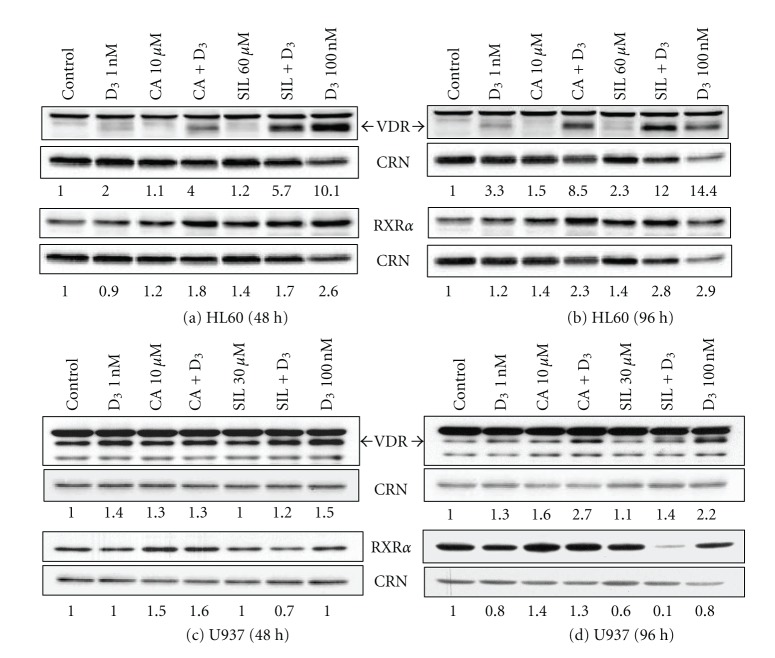
Effects of 1,25D, carnosic acid and silibinin, alone and in combination, on VDR and RXR*α* protein expression in HL60 and U937 cells. Cells were treated for 48 h (1 × 10^5^ cells/mL; (a), (c)) or 96 h (4 × 10^4^ cells/mL; (b), (d)) with 0.1% ethanol (control) or the indicated test agents. Protein expression was then determined by Western blotting. Calreticulin (CRN) was used as the protein loading control. Representative blots of at least 3 independent experiments are shown.

We then performed Western blot analysis of VDR and RXR*α* protein expression in untransfected HL60 and U937 cells following incubations with 1,25D, polyphenols, and their combinations for 48 h and 96 h. The data demonstrated that 1,25D alone induced a dose- and time-dependent elevation of VDR levels in both HL60 and U937 cells (Figures 4(a–d)) and of RXR*α* levels in HL60 cells (Figures 4(a) and 4(b)). When added alone, CA and SIL caused only modest increases in VDR levels in both cell lines but cooperated with 1 nM 1,25D in this effect. This cooperative VDR upregulation tended to strengthen with time and was much stronger in HL60 cells than in U937 cells (compare panels (a), (b) and (c), (d) in Figure 4). A similar, though less pronounced, positive cooperation between both polyphenols and 1,25D was also observed for RXR*α* levels in HL60 cells. However, in U937 cells, only CA, alone or in combination with 1,25D, positively affected RXR*α* expression. In contrast, treatment with SIL or 1,25D alone tended to decrease RXR*α* levels with time (Figures 4(c) and 4(d)) and their combination caused a marked time-dependent RXR*α* downregulation in these cells (Figures 4(c) and 4(d)). The latter inhibitory effect may, at least in part, account for the attenuated VDRE transactivation observed in 1,25D/SIL-treated U937 cells (Figure 3(b)).

### 3.4. Silibinin in Combination with 1,25D Downregulates RXR*α* mRNA Expression and Decreases RXR*α* Protein Stability in U937 Cells

**Figure 6:**
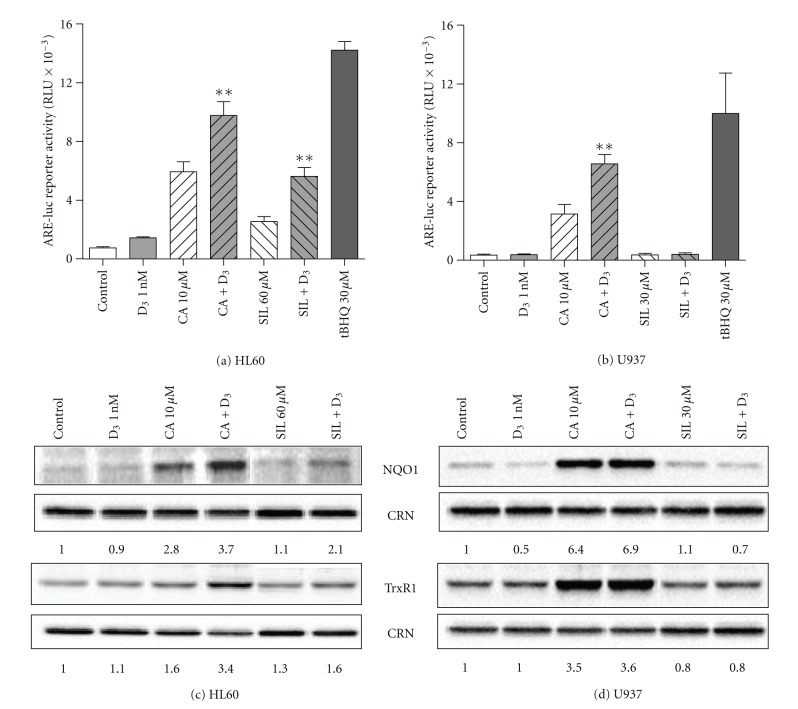
Silibinin promotes downregulation of RXR*α* in U937 cells but not in HL60 cells. (a), (b) HL60 and U937 cells (5 × 10^4^ cells/mL) were treated with 0.1% ethanol (control) or the indicated test agents, for 24 h. RXR*α* mRNA expression was determined by quantitative RT-PCR. The data are the means ± SE of 3 independent experiments performed in duplicate. ^♦^ *P* < 0.05, combination versus control.(c) U937 cells (5 × 10^4^ cells/mL) were preincubated for 72 h with 0.1% ethanol (lanes 1–6), silibinin alone (lanes 7–9), or in combination with 1,25D (lanes 10–12) followed by treatment with vehicle (H_2_O, lanes 1–3) or cycloheximide (CHX, lanes 4–12) for 0–8 h, as indicated. Cells were then lysed and protein levels were determined by Western blotting. Calreticulin (CRN) was used as the protein loading control. Representative blots of 3 independent experiments are shown. (d) Graphical representation of changes in RXR*α* protein levels (determined as shown in panel (c)) relative to 0 h (lanes 1, 4, 7, 10, resp., for corresponding treatments); means ± SE; *n* = 3. ^ ♦^ *P* < 0.05.

To determine the mode by which treatments with SIL and its combination with 1,25D reduce RXR*α* protein levels in U937 cells, we first compared the effects of SIL, CA, 1,25D, alone and together, on RXR*α* mRNA expression in both HL60 and U937 cells. As shown in Figure 6(a), neither treatment significantly affected RXR*α* mRNA levels in HL60 cells following 24-h incubations. A similar pattern was observed in U937 cells for all of the treatments except for the 1,25D/SIL combination which markedly lowered RXR*α* mRNA expression levels compared to either untreated cells or treatments with single agents. The latter effect correlated with a strong reduction in RXR*α* protein levels following 1,25D/SIL treatment (e.g., Figure 4(d)). To examine if this reduction can also result from a decrease in RXR*α* protein stability, we performed cycloheximide chase experiments. U937 cells were preincubated for 24–72 h with or without SIL or SIL + 1,25D, washed, and exposed to 400 *μ*M cycloheximide [22] to block protein synthesis. The time course of RXR*α* protein degradation in different samples was then followed for up to 8 h by Western blot analysis of relative protein levels. Cycloheximide did not significantly affect cell viability during the treatment. Interestingly, as shown in Figures 6(c) and 6(d), higher rates of reduction in RXR*α* levels were obtained in cells pretreated for 72 h with SIL alone (Figure 6(c), lanes 7–9) and, particularly, in combination with 1,25D (Figure 6(c), lanes 10–12), as compared to control cells (Figure 6(c), lanes 4–6). However, shorter preincubations revealed either less pronounced (48 h) or no (24 h) appreciable effects of SIL or SIL + 1,25D on RXR*α* degradation rates in the presence of cycloheximide (data not shown). Therefore, it appears that the time-dependent reduction in RXR*α* protein levels following treatment of U937 cells with SIL and, especially, its combination with 1,25D (Figures 4(c) and 4(d)), may result from both the earlier inhibition of mRNA expression (Figure 6(b)) and later decrease in protein stability (Figures 6(c) and 6(d)).

### 3.5. Silibinin Activates the Nrf2/Antioxidant Response Element (Nrf2/ARE) Signaling Pathway in HL60 Cells but Not in U937 Cells

**Figure 5:**
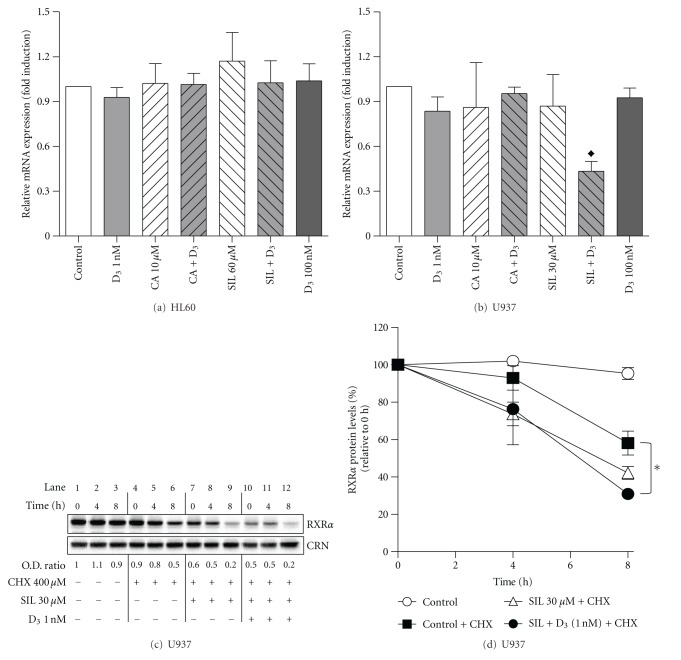
Effects of carnosic acid, silibinin, and 1,25D, alone and in combination, on the Nrf2/ARE signaling pathway in HL60 and U937 cells. (a), (b) Cells were transiently transfected with 4xARE-luc and *Renilla* luciferase reporter constructs followed by treatment with 0.1% ethanol (control) or indicated test agents for 24 h. *tert*-Butylhydroquinone (tBHQ), a classical Nrf2/ARE activator [23], was used as the positive control. The relative 4xARE-luc activity (means ± SE) was calculated from the data of 4 individual experiments performed in triplicate. **P < 0.01, combination *versus* sum of single agents. (c), (d) Cells (1 × 10^5^ cells/mL) were treated with 0.1% ethanol (control) or the indicated test agents, for 48 h. Protein expression was then determined by Western blotting. Calreticulin (CRN) was used as the protein loading control. Representative blots of at least 3 independent experiments are shown.

Our recent data have demonstrated that CA and 1,25D can synergistically activate the Nrf2/ARE signaling pathway and that Nrf2 acts as an upstream positive regulator of VDR and RXR*α* expression in U937 cells [11]. We, thus, determined whether SIL, alone or together with 1,25D, is capable of activating this pathway in HL60 and U937 cells, as compared with CA ± 1,25D. Using ARE-Luc reporter gene assays in transiently transfected cells, we found that both CA and, to a lesser extent, SIL induced ARE transactivation in HL60 cells and that the addition of 1 nM 1,25D, which alone had only a slight effect, synergistically potentiated the effects of the two polyphenols (Figure 5(a)). Likewise, both CA and SIL as well as 1,25D/CA and 1,25D/SIL combinations induced the expression of the Nrf2/ARE-responsive gene products, NAD(P)H quinone oxidoreductase-1 (NQO1) and thioredoxin reductase-1 (TrxR1), in these cells to different extent (Figure 5(c)). On the other hand, only CA, alone or together with 1,25D, was capable of trasactivating the ARE reporter (Figure 5(b)) and inducing NQO1 and TrxR1 expression, whereas SIL ± 1,25D even tended to decrease the levels of these proteins (Figure 5(d)).

## 4. Discussion

In this study we characterized the distinct modulatory effects of SIL on myeloid differentiation of HL60 and U937 human AML cells induced by a low, near physiologic concentration of 1,25D. For comparison, we utilized another plant polyphenol, CA, which differentiation-enhancing activity has been consistently demonstrated in both cell lines [7–9, 11, 20]. SIL had also been first described as phytochemical which can synergistically potentiate 1,25D-induced differentiation of HL60 myeloblastic leukemia cells [15] and this finding was later confirmed in our studies [7, 20]. However, when testing the promonocytic leukemia cell lines U937 and THP-1, which can also be induced to differentiate by 1,25D, we observed a surprising inhibition of the 1,25D effect by this polyphenol ([16] and this study). This inhibition seems to be a rather unusual phenomenon in view of a number of reports consistently showing differentiation-enhancing activity of various plant-derived bioactive compounds. Among these are different polyphenols (besides CA and SIL) [7, 24–26], sesquiterpene lactones [27–29], carotenoids [30, 31], and other phytochemicals, such as genistein [32], capsaicin [33], or cotylenin A [34].

Mechanisms underlying the potentiating effects of the above compounds on 1,25D-induced differentiation of AML cells have been extensively studied and shown to involve the activation of various signaling kinases, such as protein kinase C [15, 28, 29], phosphatidylinositol 3-kinase [28]; MAPKs, including ERK [7, 15, 28, 29] and JNK [20], as well as the transcription factors AP-1 [7, 11, 20], Egr-1 [8, 20], and Nrf2 [11]. Furthermore, the differentiation-enhancing effects of some phytochemicals, for example, curcumin [24] and parthenolide [27], or unrelated antioxidants, for example, *α*-tocopherol succinate [30], were associated with their inhibition of NF*κ*B, the transcription factor which has been reported to antagonize VDR-mediated 1,25D actions [35, 36].

Interestingly, activation of at least some of the above signaling and transcriptional pathways has been shown to promote VDR, and RXR*α* expression, which may eventually represent a major cause for the sensitization of cancer cells to lower doses of 1,25D in the presence of phytochemicals or other enhancers of 1,25D action. For instance, activation of the p38 and JNK MAPK pathways in breast cancer cells was found to increase VDR expression via upregulation of AP-1 which can bind to and transactivate the VDR gene promoter [37]. In addition, AP-1 was found to play an important role in regulation of RXR*α* expression in osteoblastic cells [38]. Our recent study has demonstrated that in U937 cells the expression of AP-1, VDR, and RXR*α* can be controlled by Nrf2 activity [11]. Correspondingly, we have previously shown that the synergistic differentiation effects of 1,25D and CA in AML cells correlated with a marked cooperative increase in VDR and RXR*α* levels [8, 11]. A similar VDR protein upregulation in leukemia cells was observed when 1,25D was combined with other inducers or enhancers of differentiation, such as all trans retinoic acid (ATRA) [39] and bufalin [40], a major digoxin-like component of toad venom. Likewise, enhanced antiproliferative effects of 1,25D combinations with the soy isoflavone genistein [41–43] or the *RRR* stereoisomer of *α*-tocopherol [44] in nonleukemic cancer cells were associated with elevated VDR expression.

In view of the above findings we hypothesized that the mechanism by which SIL attenuates 1,25D-induced differentiation in U937 cells could result from a decrease in VDR/RXR*α* expression and/or activity. Indeed, we obtained several lines of evidence indicating a strong correlation between the attenuated response to 1,25D and reduced vitamin D receptor activity, namely, the SIL inhibition of 1,25D-induced VDRE transactivation that was consistent with a marked decrease in RXR*α* expression levels and protein stability. Of note, SIL also inhibited ATRA-induced differentiation of U937 cells in our experiments (manuscript in preparation). Since the retinoic receptor alpha (RAR*α*) heterodimerizes with RXR*α* to produce a functionally active receptor for ATRA (e.g., [45]), these data suggest that RXR*α* can serve as a common target for the negative effect of SIL on maturation of these cells induced by both 1,25D and ATRA. Surprisingly, in contrast to HL60 cells, 1,25D itself tended to downregulate RXR*α* expression in U937 cells and, thus, the greatest reduction in RXR*α* levels was observed in cells treated with the 1,25D/SIL combination. One possible explanation for the “anti-differentiation” behavior of SIL in these cells is that it may activate some differentiation-antagonizing factor(s) and cooperate with 1,25D in this effect. Indeed, it has been shown that 1,25D and SIL can concurrently upregulate and activate ERK5 (big MAPK-1) and its upstream regulator, Cot1 kinase [16], which has a negative effect on 1,25D-induced differentiation of AML cells [46]. In addition, high expression levels of at least a few transcription factors, for example, lymphoblastic-leukemia-derived sequence 1 (LYL1) [47] and homeobox B6 (HOXB6) [48], were associated with inhibition of monocytic differentiation of myeloid precursors. Although it was found that ERK5 does not directly phosphorylate RXR*α* in response to 1,25D [49], the relationship between the other negative regulators of monocytic differentiation and RXR*α* expression or function remains to be elucidated. Since Nrf2 may function as a positive regulator of RXR*α* expression [11], the failure of SIL to stimulate the Nrf2/ARE in U937 cells may contribute to its negative effect on RXR*α* levels and, thereby, on 1,25D-induced differentiation of these cells. The reason why, in contrast to CA, SIL can activate Nrf2/ARE in HL60 cells but not in U937 cells is unclear and may be related to possible differences in the metabolic fate of SIL in the two cell types.

In conclusion, our results demonstrate that the contrasting effects of SIL on the differentiation of myeloblastic (HL60) and promonocytic (U937) AML cells are related, at least in part, to its opposing modulation of the VDR signaling pathway. We suggest that U937 cells may present a model for patient-derived leukemic blasts which respond to SIL [16, 17] and similarly acting compounds by inhibition of 1,25D-induced differentiation. SIL has been shown to inhibit proliferation of various types of cancer cells (see [50, 51] for recent reviews). Likewise, this polyphenol demonstrated a marked antiproliferative activity in both AML cell lines tested here, even though it negatively affected the differentiation of U937 cells. Therefore, further preclinical studies are required to determine the potential use of different plant polyphenols for AML treatment and/or prevention.
